# Surgical treatment of ductal biliary recurrence of poorly cohesive gastric cancer mimicking primary biliary tract cancer: a case report

**DOI:** 10.1093/jscr/rjac132

**Published:** 2022-04-11

**Authors:** Edoardo Poletto, Andrea Ruzzenente, Giulia Turri, Simone Conci, Serena Ammendola, Claudio Luchini, Aldo Scarpa, Alfredo Guglielmi

**Affiliations:** Division of General and Hepato-Pancreato-Biliary Surgery, Department of Surgery, University of Verona, Verona, Italy; Division of General and Hepato-Pancreato-Biliary Surgery, Department of Surgery, University of Verona, Verona, Italy; Division of General and Hepato-Pancreato-Biliary Surgery, Department of Surgery, University of Verona, Verona, Italy; Division of General and Hepato-Pancreato-Biliary Surgery, Department of Surgery, University of Verona, Verona, Italy; Division of Pathologic Anatomy and Histology, Department of Pathology and Diagnostics, University of Verona, Verona, Italy; Division of Pathologic Anatomy and Histology, Department of Pathology and Diagnostics, University of Verona, Verona, Italy; Division of Pathologic Anatomy and Histology, Department of Pathology and Diagnostics, University of Verona, Verona, Italy; Division of General and Hepato-Pancreato-Biliary Surgery, Department of Surgery, University of Verona, Verona, Italy

**Keywords:** gastric cancer, metastasis, biliary tract neoplasms, pancreato-duodenectomy, case report

## Abstract

Ductal biliary recurrence of cancers arising in other anatomical districts is a rare event, usually observed in the setting of disseminated disease; hence surgery is rarely a viable option. We present the case of a 56-year-old male who underwent subtotal gastric resection 7 years earlier for a poorly cohesive gastric cancer, presenting with obstructive jaundice. Magnetic resonance imaging and computed tomography scan suggested primary malignant obstruction of the main bile duct. Percutaneous transhepatic biliary drainage was performed to palliate jaundice and obtain biopsies; pathological examination suggested a ductal biliary recurrence of gastric carcinoma. Pancreaticoduodenectomy and bile duct resection were performed. Histology, immunohistochemistry and molecular profiling confirmed that the stenosis represented a gastric cancer metastasis. This is the first case of an isolated ductal biliary recurrence of gastric cancer amenable to surgical resection. This clinical case suggests that biliary obstructions in patients with previous oncological history require biliary biopsies to exclude a recurrent disease.

## INTRODUCTION

Up to 20% of all malignant biliary obstructions are due to secondary cancer, resulting from parenchymal metastases obstructing intrahepatic bile ducts, compression by enlarged lymph nodes in the hepatoduodenal ligament, local recurrence or, rarely, intraluminal invasion of main bile duct (MBD). The most frequent causes are gastric, colic, breast, kidney and lung cancers [1].

After curative resection for gastric cancer, recurrence may arise as biliary tract obstruction in 1.4–2.3% of the patients, usually due to metastatic nodes of the hepatoduodenal ligament that compress the MBD and liver metastases with intrahepatic biliary obstruction. An isolated bile duct invasion or ductal metastasis is rarely detected [1, 2].

Patients with jaundice due to recurrent gastric cancer often present with disseminated disease; hence treatments are systemic chemotherapy and jaundice palliation through percutaneous transhepatic biliary drainage (PTBD) [3, 4].

Nonetheless, isolated biliary duct recurrences imply diagnostic and therapeutic challenges and there is no evidence in literature concerning the optimal treatment.

## CASE REPORT

A 56-year-old Caucasian male was referred to our institution for the onset of obstructive jaundice with a total bilirubin of 11.97 mg/dl.

The patient was diagnosed with gastric cancer 7 years before admission. In that occasion, he underwent open subtotal gastrectomy, D2 lymphadenectomy and reconstruction by Roux-en-Y gastrojejunal anastomosis. Histopathological diagnosis was poorly cohesive, signet ring cell carcinoma, poorly differentiated and extended to the subserosa (pT3); 21 nodes retrieved from the specimen showed no sign of metastasis (N0); resection margins were not involved (R0). The patient underwent 6 months of adjuvant chemotherapy with capecitabine, and regular follow-up for 6-years negative for recurrence.

**Figure 1:**
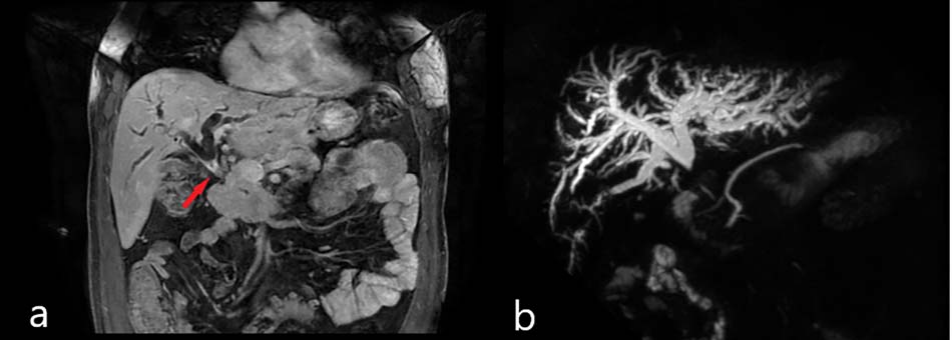
Magnetic resonance imaging (MRI): (**a**) coronal scan showing the dilatation of bile ducts and the thickening of the MBD (red arrow); (**b**) magnetic resonance cholangiopancreatography (MRCP) showing the dilatation of intrahepatic bile ducts and the absence of signal from the MBD below the confluence to the papilla of Vater.

**Figure 2:**
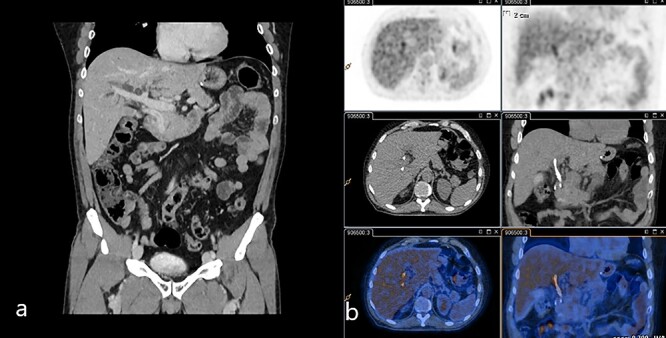
(**a**) Venous phase of the CT scan showing the dilated biliary tree and the contrast-enhanced thickening of the common bile duct. (**b**) 18-FDG-PET/CT scan showing the slight uptake of marked glucose along the common bile duct.

Abdominal US showed diffuse dilatation of the biliary tree. Magnetic resonance imaging and magnetic resonance cholangiopancreatography highlighted a 6-cm-long concentric thickening of the MBD below the biliary confluence towards the papilla of Vater (Fig. 1). Staging computed tomography (CT) showed no signs of lung or liver metastases (Fig. 2a). During percutaneous cholangiography, biopsies were performed, and a percutaneous internal-external drainage left in place to palliate jaundice. An 18-Fluorodeoxyglucoose Positron emission tomography (18-FDG-PET/CT) confirmed a slight uptake at the thickening of the MBD (Fig. 2b). Oncological markers, carcinoembryonic antigen and carbohydrate antigen 19-9 were not elevated.

Unexpectedly, the biopsies showed a ‘diffused infiltration by poorly cohesive cells, signet ring type’. Immunohistochemistry showed positivity for low-molecular weight cytokeratin 8/18/19 and intestinal marker CDX2, a profile like the gastric primary, suggesting an intraductal biliary recurrence.

Radical surgery was considered a therapeutic option given the good general conditions and limited extent of the disease. The patient underwent re-laparotomy and a wide Kocher manoeuvre with exposure of the MBD, thickened and hardened from intra-pancreatic tract to the common bile duct. The MBD was divided at the confluence and pancreato-duodenectomy with regional lymphadenectomy was performed. Post-operative course was uneventful, and the patient was discharged on the eighth post-operative day.

Pathological examination of the surgical specimen showed a poorly cohesive signet ring cell carcinoma, infiltrating the MBD wall, the duodenal wall, the peri-visceral fat tissue and the pancreatic parenchyma. Immunohistochemistry showed a diffuse positivity for Cytokeratin 8/18/19 and CDX2. Nodal metastases were found in 7 out of 19 of the nodes sampled. Since primary signet ring carcinoma of the bile duct is an extremely rare diagnosis [5], a revision of the specimen of the previous gastrectomy was requested. Further confirmation was provided by CORE panel next-generation sequencing [6] of primary gastric and recurrent bile duct cancer. They both shared the same molecular profile in terms of driver gene mutations, definitively demonstrating that the biliary tumour was a relapse of the gastric primary (Table 1).

**Table 1:** Genomic analysis of the primitive gastric cancer and biliary recurrence: The gastric primary and the biliary tumour share the same molecular profile in terms of driver gene mutations

Tumour	TMB	MS	Gene alterations		CNV	
			Gene	Variation	Gene	Variation
Primitive gastric cancer	8,1	MSS	*BLM* *EP300* *RET*	p.A614T p.P1911del p.A513G	*ATM* *NF1*	LOH LOH
Biliary ductal recurrence	8,6	MSS	*BLM* *EP300* *RET*	p.A614T p.P1911del p.A513G	*APLNR* *CDKN1B*	Gain LOH

There were some differences in terms of copy number variation, but this is in line with well-established knowledge on the independent evolution of metastases. TMB, tumour mutational burden; MS, microsatellite status; MSS, microsatellite stability; CNV, copy number variations; LOH, loss of heterozygosity.

After multidisciplinary discussion, the patient was eligible to chemotherapy with capecitabine and oxaliplatin, which he performed for 6 months showing no sign of relapse.

## DISCUSSION

This case presented is uncommon: although the presentation suggested a primary biliary tumour, pre- and post-operative examination, integrating histology with molecular profiling, demonstrated a ductal recurrence from a previously resected gastric cancer. Moreover, disease recurrence was confined at a single site; therefore, radical surgery was deemed possible.

Isolated ductal recurrences from gastric cancer are extremely rare and only few cases are reported. In their experience, Lee *et al*. reported 54 cases of biliary obstruction due to metastatic gastric carcinoma, only 3 of them caused by ductal biliary recurrence [2]. Gwon *et al*. reported 67 cases of ductal biliary recurrence from gastric cancer, all treated with PTBD and metallic stenting placement [3]. Lee *et al*. reported 24 cases, 6 of them histologically proven, treated with good results with metallic stent placement after PTBD. Lee *et al*. also described the common radiological and histological features of this type of recurrence: a uniform, linear/band-like, enhanced biliary wall thickening and malignant cells scattered in the submucosal layer with desmoplastic reaction without disruption of the epithelial layer [7]; our patient showed a compatible radiological and histological patterns.

Median time between surgery for gastric cancer and biliary recurrence reported range between 10 and 22 months [1–96.8 months] [8, 9]. Shorter time to recurrence may indicate a poorer prognosis [8].

Our case is peculiar since histologically proven biliary ductal recurrence appeared after a long disease-free period (84 months) as a single-site recurrence.

Based on previous medical history, pre-operative biopsies were obtained to exclude other diseases causing biliary stenosis (primary biliary malignancy, autoimmune biliary disease). Biopsies showed a poorly cohesive cell carcinoma, which can be rarely observed in biliary tract cancers: only 11 cases of histologically proven primary signet ring cell carcinoma of the MBD have been published [5].

By integrating histology, immunohistochemistry and molecular profile, a clear demonstration of the metastatic nature of the recurrent tumour was provided. Correct identification of local/distant relapse versus a new primary represents an important step in the management of patients with recurrent tumours, above in the pancreatobiliary district where the timeframe for surgery is usually very short for the biological aggressiveness of the most common cancer types [10].

To the best of our knowledge this is the first published case of curative surgery with radical intent of recurrent gastric cancer mimicking primary biliary cancer. This clinical case suggests that a biliary obstruction in patients with previous oncological history should require biliary biopsies in all cases to exclude a recurrent disease.

## DATA AVAILABILITY

The clinical data of this case report are available, after proper anonymization, for the Editor to review.

## AUTHOR CONTRIBUTIONS

A.R., E.P. and C.L. all made substantial contribution to the manuscript conception and drafting. All the authors have been involved in drafting and revising the content of the manuscript. All authors read and approved the final manuscript.

## CONSENT FOR PARTICIPATION AND PUBLICATION

Written informed consent for the gathering and use of clinical data and its publication was obtained from the patient. A copy of said consent is available for review by the Editor of this article.

## CONFLICT OF INTEREST STATEMENT

Prof. Andrea Ruzzenente, Dr Edoardo Poletto, Dr Giulia Turri, Dr Simone Conci, Dr Serena Ammendola, Dr Claudio Luchini, Prof. Aldo Scarpa and Prof. Alfredo Guglielmi have no conflict of interest or financial ties to disclose.

## FUNDING

This case report did not receive any specific grant from funding agencies in the public, commercial or not-for-profit sectors.
